# Exploring fluoroquinolone resistance mechanisms and the effects of carbonyl cyanide 3-chlorophenylhydrazone (CCCP) in *Acinetobacter baumannii*

**DOI:** 10.3389/fmed.2025.1527662

**Published:** 2025-05-09

**Authors:** Mohsen Nazari, Mohammad Sina Alikhani, Jaber Hemmati, Amjad Ahmadi, Seyyed Hamid Hashemi, Mohammad Yousef Alikhani

**Affiliations:** ^1^Department of Microbiology, School of Medicine, Hamadan University of Medical Sciences, Hamadan, Iran; ^2^Student Research Committee, Urmia University of Medical Sciences, Urmia, Iran; ^3^Infectious Disease Research Center, Avicenna Institute of Clinical Sciences, Hamadan University of Medical Sciences, Hamadan, Iran

**Keywords:** *Acinetobacter baumannii*, fluoroquinolone, efflux pump, resistance, carbonyl cyanide

## Abstract

**Objectives:**

This study aims to investigate the prevalence and mechanisms of fluoroquinolone resistance in *Acinetobacter baumannii* strains isolated from hospitals in Hamadan, west of Iran. It investigates the role of specific resistance genes and mutations in contributing to this resistance. In addition, the effects of carbonyl cyanide 3-chlorophenylhydrazone (CCCP) on the susceptibility of *A. baumannii* to fluoroquinolones will be evaluated to identify potential strategies to combat this growing problem.

**Methods:**

A total of 102 *A. baumannii* isolates were collected from various clinical specimens between February and August 2023. Antimicrobial susceptibility testing was performed using the Kirby-Bauer disk diffusion method according to CLSI guidelines, focusing on eight antibiotics, including ciprofloxacin and levofloxacin. Minimum inhibitory concentration (MIC) and minimum bactericidal concentration (MBC) evaluations were also performed for these fluoroquinolones. The presence of plasmid-dependent fluoroquinolone resistance (PMQR) genes and mutations in the gyrA and parC genes were assessed by PCR. The effect of CCCP on antibiotic susceptibility and expression of efflux pump encoding gene was evaluated by real-time PCR.

**Results:**

The study revealed alarmingly high resistance rates among the 102 *A. baumannii* isolates, with 97% resistant to imipenem, 96% to gentamicin, 92% to ciprofloxacin, and 86% to levofloxacin. Of the isolates, 87 were classified as multidrug resistant (MDR). Several resistance genes were identified, including *qnrS* (77.45%), *oqxA* (73.52%), and *qnrA* (72.54%). Mutations in the *gyrA* and *parC* genes were detected in several isolates, contributing to the observed resistance. In addition, treatment with CCCP resulted in a significant reduction in MICs for both ciprofloxacin and levofloxacin, highlighting its potential role in mitigating resistance.

**Conclusion:**

The findings underscore the urgent need for improved surveillance and treatment strategies due to the high prevalence of fluoroquinolone resistance. While CCCP demonstrated potential in restoring antibiotic susceptibility, further studies are needed to assess its clinical applicability and potential toxicity. Additionally, the study is limited by its focus on a single geographic region, and further investigations across broader populations are necessary to generalize these findings.

## 1 Introduction

*Acinetobacter baumannii* is a notorious opportunistic pathogen, frequently responsible for infections in hospitalized patients, particularly in intensive care units (ICUs) (1). Although it generally exhibits low virulence, *A. baumannii* can cause severe infections, often through contaminated respiratory equipment and catheters (2, 3). These infections include skin and soft tissue infections, wound infections, urinary tract infections, secondary meningitis, and bloodstream infections such as bacteremia (4). Among these, ventilator-associated pneumonia and bacteremia are particularly common, with ventilator-associated pneumonia mortality rates ranging from 40% to 70% (5). Bloodstream infections have mortality rates between 28% and 43%, while urinary tract infections, typically related to prolonged catheter use, are more common in general hospital wards (6). Wound infections are often observed in patients with severe burns or deep combat-related injuries (7). High-risk groups include the elderly, immunocompromised individuals, and those exposed to long hospital stays, colonized patients, or broad-spectrum antibacterial treatments (8, 9).

A major challenge in managing *A. baumannii* infections is the emergence of multidrug-resistant (MDR) strains (10). These strains exhibit resistance to multiple antibiotic classes, including beta-lactams, aminoglycosides, and fluoroquinolones, largely due to genes located on mobile genetic elements such as transposons and integrons (11). Carbapenem antibiotics like imipenem and meropenem are commonly used to treat *A. baumannii* infections, but resistance to these drugs ranges from 28% to 85% (12).

Fluoroquinolones, which inhibit bacterial DNA replication by targeting DNA gyrase and topoisomerase IV, are key in treating *A. baumannii* infections (13). However, resistance to fluoroquinolones is primarily caused by chromosomal mutations in the quinolone resistance-determining regions (QRDR) of the gyrA and parC genes, leading to alterations in the enzymes responsible for DNA replication (14). The most commonly reported mutations associated with fluoroquinolone resistance occur at Ser-83 in the gyrA gene and Ser-80 in the parC gene. In contrast, genetic alterations at the gyrB (Ser83Leu) and parE (Ser80Leu) loci are not implicated in fluoroquinolone resistance (15). Additionally, plasmid-mediated quinolone resistance (PMQR) genes provide an additional mechanism of resistance, often complementing chromosomally encoded mutations (16). PMQR mechanisms include qnr peptides that protect topoisomerases and efflux pumps such as QepA and OqxAB, which actively expel fluoroquinolones from bacterial cells (14, 17).

Despite extensive studies on fluoroquinolone resistance in *A. baumannii*, there remains a critical gap in understanding the potential for restoring antibiotic susceptibility through efflux pump inhibition. Efflux pumps significantly contribute to resistance by expelling antibiotics before they reach their intracellular targets, thereby reducing their effectiveness (18, 19). Carbonyl cyanide 3-chlorophenylhydrazone (CCCP) is a known efflux pump inhibitor that disrupts the proton motive force across bacterial membranes, potentially restoring antibiotic efficacy (20, 21). However, while CCCP has been explored in other bacterial species, its role in reversing fluoroquinolone resistance in *A. baumannii* remains underexplored.

This study aims to explore the prevalence and mechanisms of fluoroquinolone resistance in *A. baumannii* strains isolated from hospitals in Hamadan, as well as the effects of CCCP on the susceptibility of *A. baumannii* to fluoroquinolones.

## 2 Materials and methods

### 2.1 Patients and specimens

From February to August 2023, 102 *A. baumannii* isolates were obtained from different wards in teaching hospitals in Hamadan, west of Iran. These isolates were obtained from different clinical samples, including broncho-alveolar lavage, sputum, intravascular catheters, and cerebrospinal fluid (CSF), and wound swabs. Inclusion criteria required isolates to be obtained from patients with confirmed infections, as determined by clinical and laboratory findings, while environmental or duplicate samples from the same patient were excluded. The *Acinetobacter* genus was chemically identified by oxidative-fermentative (OF), oxidase-catalase, motility, and triple sugar iron (TSI) tests, along with microscopic analysis. Genetic confirmation of *A. baumannii* was performed by PCR detection of the *bla_*oxa*_51*-like gene, and the resulting gene products were sent for sequencing (22).

This study was approved by the Ethics Committee of Hamadan University of Medical Sciences, Hamadan, Iran (Ethical approval No. IR.UMSHA.REC.1401.972). Written informed consent was obtained from all subjects and/or their legal guardians. All methods were conducted in accordance with relevant guidelines and regulations. We reported our findings according to the STROBE guidelines.

### 2.2 Antimicrobial susceptibility testing

Antimicrobial susceptibility testing was conducted using the Kirby-Bauer disk diffusion technique, following the 2024 guidelines from the Clinical and Laboratory Standards Institute (CLSI) (23). Testing was performed on Mueller-Hinton agar (MHA) plates with a selection of eight antibiotic disks: gentamicin (GM), minocycline (MN), ciprofloxacin (CIP), ampicillin-sulbactam (SAM), levofloxacin (LEV), piperacillin-tazobactam (PTZ), ceftazidime (CAZ), and imipenem (IMP), obtained from Mast Group Ltd., Merseyside, United Kingdom (24). These antibiotics were chosen according to CLSI recommendations for treating *A. baumannii*. Quality control was ensured using *Escherichia coli* ATCC 25922 as the reference strain. Isolates resistant to at least three antibiotic classes were classified as multidrug-resistant (MDR) following established criteria (25).

### 2.3 Minimum inhibitory concentration (MIC) and minimum bactericidal concentration (MBC)

Minimum inhibitory concentration values for ciprofloxacin and levofloxacin were determined by a modified microdilution method according to CLSI guidelines for selected isolates (23). Fresh bacterial colonies were initially grown overnight in cation-adjusted mueller hinton broth (CaMHB) at 180 rpm and 37°C. This culture was then diluted to achieve a 0.5 McFarland standard, and the optical density (OD) was adjusted to 0.09, aligning with the typical range of 0.08–0.1, which corresponds to 10^8^ CFUs/mL. For the assay, a further dilution was made to reach a concentration of 10^6^ CFUs/mL. In parallel, 100 μL serial dilutions of the antimicrobials were prepared in CaMHB and dispensed into 96-well microplates. The concentrations of ciprofloxacin and levofloxacin ranged from 0.25–512 to 0.5–1,024 μg/mL, respectively. Then, 100 μL of bacterial suspension, equivalent to 105 CFU/mL, was added to the wells containing the diluted antimicrobials. Plates were incubated overnight at 37°C and the MIC was defined as the lowest concentration that completely inhibited visible bacterial growth. Based on CLSI breakpoints, ciprofloxacin is categorized as susceptible when the MIC is below 1 μg/mL, intermediately susceptible between 1 and 4 μg/mL, and resistant if exceeding 4 μg/mL. Levofloxacin is considered susceptible at an MIC below 2 μg/mL, intermediately susceptible between 2 and 8 μg/mL, and resistant if above 8 μg/mL. The minimum bactericidal concentration (MBC) was defined as the lowest concentration of ciprofloxacin or levofloxacin capable of eliminating 99.9% of the bacterial population (26).

### 2.4 PCR detection of plasmid-dependent fluoroquinolone resistance genes (PMQR)

Colonies cultured on MHA underwent DNA extraction using a modified boiling protocol (27). In brief, a loopful of each strain was suspended in 200 μL of sterile distilled water and mixed by vortexing. The mixture was then centrifuged at 10,000 rpm for 12 min at 4°C, after which the supernatant was removed. The pellets were resuspended in 200 μL of distilled water, boiled for 12 min, and centrifuged again at 10,000 rpm for 10 min. The supernatant was collected and stored at −20°C as the DNA template for PCR amplification. PMQR genes (*qnrA*, *qnrB*, *qnrC*, *qnrS*, *qepA*, *oqxA* and *oqxB*) were screened in all isolates using specific primers in a PCR assay (Table 1).

**TABLE 1:** Primer sequences for plasmid-mediated fluoroquinolone resistance genes (PMQR) and PCR-restriction fragment length polymorphism (PCR-RFLP) analysis.

Primer name	Primer sequence (5′ to 3′)	PCR product size (bp)	References
*qnrA* m-F	AGAGGATTTCTCACGCCAGG	580	(53)
*qnrA* m-R	TGCCAGGCACAGATCTTGAC		
*qnrB* m-F	GGMATHGAAATTCGCCACTG	264	(53)
*qnrB* m-R	TTTGCYGYYCGCCAGTCGAA		
*qnrS* m-F	GCAAGTTCATTGAACAGGGT	428	(53)
*qnrS* m-R	TCTAAACCGTCGAGTTCGGCG		
*qnrC*-F	GGGTTGTACATTTATTGAATC	447	(54)
*qnrC*-R	TCCACTTTACGAGGTTCT		
*qepA*-GF	ACATCTACGGCTTCTTCGTCG	502	(55)
*qepA*-GR	AACTGCTTGAGCCCGTAGATC		
*oqxA-*F	CTCTCCTTTCTGCTCGTCGG	313	(56)
*oqxA-*R	AATAGGGGCGGTCACTTTGG		
*oqxB-*F	TAGTGCTGGTGGTGCTGGTA	489	(38)
*oqxB-*R	GGGTAGGGAGGTCTTTCTTCG		
*parC-*F	AAACCTGTTCAGCGCCGCATT	480	(38)
*parC*-R	AAAGTTGTCTTGCCATTCACT		
*gyrA*-F	AAATCTGCCCGTGTCGTTGGT	343	(57)
*gyrA*-R	GCCATACCTACGGCGATACC		
*gyrB*	GAAATGACCCGCCGTAAA	490	(44)
*gyrB*	ACGACCGATACCACAGCC		

### 2.5 Detection of gyrA and parC mutations by PCR-restriction fragment length polymorphism (PCR-RFLP)

To analyze mutations in the *gyrA* and *parC* genes, these genes were amplified with specific primers (Table 1) from the isolates showing resistance to ciprofloxacin and levofloxacin. PCR amplification products were subjected to restriction analysis using the HinfI restriction enzyme (Thermo SCIENTIFIC^®^ cat no. # ER0801). The reaction mixture consisted of 2 μL of HinfI enzyme, 10 μL of PCR products, 2 μL of 10X Buffer Tango, and 18 μL of nuclease-free water. The resulting restriction-digested DNA fragments were separated by 2% agarose gel electrophoresis and visualized under ultraviolet light. A 100 bp DNA ladder (Thermo Scientific, Waltham, MA, United States) was used to determine product sizes. For the *gyrA* gene, the PCR band size was 343 bp, and after digestion with HinfI, the presence of two bands—291 bp and 52 bp—indicated an unchanged QRDR region. Similarly, for the *parC* gene, the band size was 327 bp, and after digestion, two bands of 206 bp and 121 bp indicated an intact QRDR region (28).

### 2.6 Addition of carbonyl cyanide 3-chlorophenylhydrazone (CCCP)

The effect of CCCP on antibiotic susceptibility was evaluated for ciprofloxacin and levofloxacin. To each plate containing CaMHB, antibiotic concentrations were added in a range from 0.25 to 512 μg/mL. Following this, CCCP (obtained from Sigma-Aldrich, St. Louis, MO, United States) was introduced to each plate, bringing the final volume to 100 μL. The MIC for each antibiotic was subsequently determined with CCCP present. According to established benchmarks in prior research, a significant reduction in MIC was defined as a decrease by 4-fold or more following CCCP addition (29, 30).

### 2.7 Effect of fluoroquinolone and carbonyl cyanide 3-chlorophenylhydrazone (CCCP) on the expression of efflux pumps encoding genes

Fluoroquinolone-resistant strains possessing the *qepA* and *oqxA* genes were selected for further analysis using real-time PCR to assess the expression of the efflux pump encoding genes, *qepA* and *oqxA*. These selected strains were exposed to sub-MIC concentrations of ciprofloxacin and levofloxacin, either alone or in combination with sub-MIC concentrations of CCCP (27). The next day, total RNA was extracted using the RNX-plus Mini Kit (Sinaclon, Iran) according to the manufacturer’s instructions. The concentration, purity and integrity of the extracted RNA were evaluated. Subsequently, 1 μg of RNA was used for cDNA synthesis using an RT-PCR kit according to the manufacturer’s protocol. For real-time PCR, 2X Q-PCR Master Mix was used with 2 μL of cDNA and 1 μL of each primer for *qepA*, *oqxA*, and 16S rRNA in a final volume of 20 μL. Gene expression analysis was performed on a LightCycler^®^ 96 instrument (Roche, United States). The relative expression of target genes was calculated using the Ct method, with 16S rRNA as an internal control for each bacterial isolate.

### 2.8 Statistical analysis

Statistical analyses were conducted using GraphPad Prism 9 software (GraphPad Software, Inc., La Jolla, CA, United States). The normality of the data distribution was assessed using the Shapiro-Wilk test, while Levene’s test was applied to evaluate homoscedasticity (equal variance) across groups. Based on these assessments, parametric tests were used when data met normality and homogeneity assumptions, and nonparametric alternatives were applied otherwise. The expression levels of *qepA* and *oqxA* genes were compared using *t*-tests and chi-squared tests (χ^2^). A 95% confidence level was used in all evaluations, and results with a *p*-value of less than 0.05 were considered statistically significant. Nonlinear regression analysis was used to evaluate the relationship between the concentrations tested and the percentage of activity. All experiments were performed in triplicate.

## 3 Results

### 3.1 Isolation of *A. baumannii* from various clinical samples

A total of 102 clinical isolates were obtained from different hospital wards. The distribution of isolates was as follows: 28.43% from the Emergency ICU (29/102), 26.47% from the General ICU (27/102), 22.54% from the Poisoning ICU (23/102), 17.64% from the Neurosurgery ICU (18/102), 2.94% from the Infectious Disease Ward (3/102), and 1.96% from the Neurosurgery Ward (2/102). Of these, 57.84% (59/102) were from male patients and 42.15% (43/102) were from female patients. Regarding clinical specimen sources, 37.25% (38/102) of isolates were obtained from broncho-alveolar lavage, followed by 25.49% (26/102) from wound swabs, 23.52% (24/102) from intravascular catheters, 9.80% (10/102) from sputum, and 3.92% (4/102) from cerebrospinal fluid.

### 3.2 Antibiotic resistance pattern in *A. baumannii* isolates

Of the 102 *A. baumannii* isolates obtained from different clinical specimens, the resistance rates were as follows 97 to imipenem, 96 to gentamicin, 92 isolates were resistant to ciprofloxacin, 86 to levofloxacin, 86 to ceftazidime, 85 to piperacillin-tazobactam, 57 to ampicillin-sulbactam, and 32 to minocycline. Imipenem had the highest resistance rate, while minocycline had the lowest. A total of 87 isolates were identified as MDR.

### 3.3 MICs and MBCs

The results showed that ciprofloxacin and levofloxacin inhibited the growth of *A. baumannii* isolates with MIC values ranging from 1 to 512 μg/mL for ciprofloxacin and 2 to 512 μg/mL for levofloxacin. In addition, both antibiotics exhibited bactericidal activity against the isolates tested, with MBC values ranging from 2 to 1,024 μg/mL for ciprofloxacin and 4 to 1,024 μg/mL for levofloxacin. The geometric mean MIC values for ciprofloxacin and levofloxacin in *A. baumannii* were 41.42 and 49.77 μg/mL, respectively, and the geometric mean MBC values for ciprofloxacin and levofloxacin were consistent at 85.72 and 109.48 μg/mL, respectively (Table 2).

**TABLE 2:** MIC and MBC of ciprofloxacin and levofloxacin against *Acinetobacter baumannii* isolates, including MIC values with CCCP supplementation.

Antibiotic concentration (μg/mL)	Number of isolates (CIP MIC)	Number of isolates (CIP MBC)	Number of isolates (CIP + CCCP MIC)	Number of isolates (LEV MIC)	Number of isolates (LEV MBC)	Number of isolates (LEV + CCCP MIC)
0.5	0	0	2	0	0	0
1	4	0	6	0	0	5
2	6	4	9	12	0	10
4	4	6	11	4	10	10
8	6	3	4	3	5	4
16	11	7	12	8	3	10
32	17	8	16	14	9	11
64	16	22	18	13	9	16
128	18	13	17	22	18	21
256	15	17	7	22	19	15
512	5	17	0	4	23	0
1024	0	5	0	0	6	0

CIP, ciprofloxacin; LEV, levofloxacin; MIC, minimum inhibitory concentration; MBC, minimum bactericidal concentration; CCCP, inhibidor carbonyl cyanide 3-chlorophenylhydrazone.

### 3.4 The distribution pattern of PMQR

**FIGURE 1:**
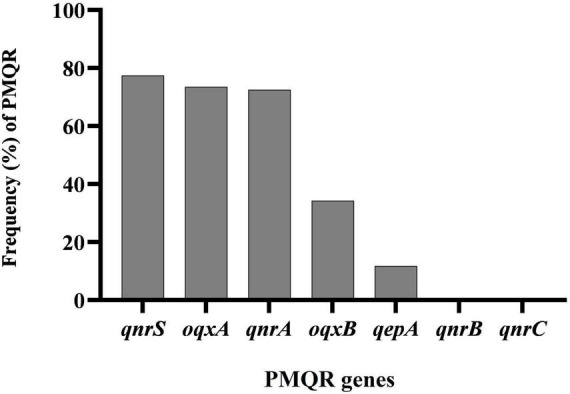
Distribution of plasmid-mediated fluoroquinolone resistance (PMQR) genes among clinical isolates of *Acinetobacter baumannii*.

In a collection of 102 *A. baumannii* isolates from various clinical samples, the frequencies of specific resistance genes were assessed. The *qnrS* gene was found in 77.45% (79 isolates), *oqxA* in 73.52% (75 isolates), *qnrA* in 72.54% (74 isolates), *oqxB* in 34.31% (35 isolates), and *qepA* in 11.76% (12 isolates). No isolates showed the presence of the *qnrB* or *qnrC* genes (Figure 1).

### 3.5 *gyrA* and *parC* mutations

In our study, 22 isolates had mutations in both the *gyrA* and *parC* genes, with the MIC for all isolates exceeding 32 μg/mL for ciprofloxacin and 64 μg/mL for levofloxacin. In addition, 38 isolates had single mutations in the *gyrA* gene, and 34 isolates had single mutations in the *parC* gene. Notably, eight isolates showed no mutations, with MIC values for levofloxacin below 8 μg/mL. Further details are given in Table 3.

**TABLE 3:** MIC distribution of ciprofloxacin and levofloxacin and *gyrA* and *parC* mutations.

Number of isolates	Mutation of *gyrA*	Mutation of *parC*	No. of CIP-resistance isolates MICs (μg/mL)								No. of LEV-resistance isolates MICs (μg/mL)						
			< 4	4	8	16	32	64	128	> 128	< 8	8	16	32	64	128	> 128
22	+	+	0	0	0	0	3	6	4	9	0	0	0	0	6	6	10
38	+	−	2	1	3	6	6	6	10	4	4	1	5	9	2	9	8
34	−	+	2	2	2	5	8	4	4	7	4	2	3	5	5	7	8
8	−	−	6	1	1	0	0	0	0	0	8	0	0	0	0	0	0

CIP, ciprofloxacin; LEV, levofloxacin; MIC, minimum inhibitory concentration.

### 3.6 Effect of CCCP on bacterial resistance

**FIGURE 2:**
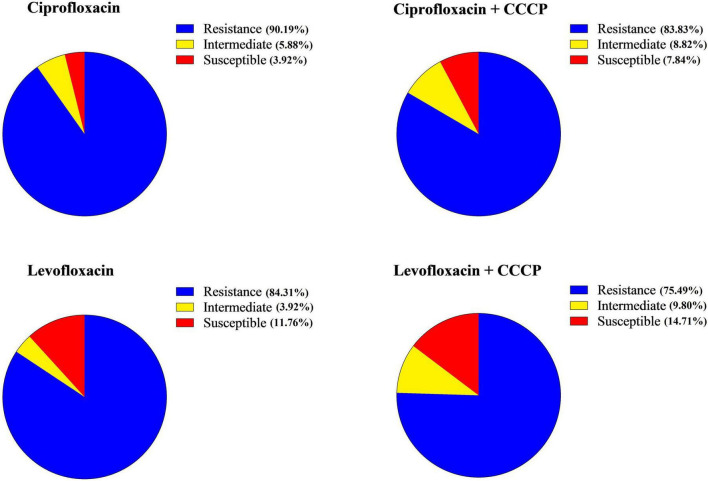
Profiles of antibiotic susceptibility for each antibiotic prior to and following the addition of carbonyl cyanide 3-chlorophenylhydrazone (CCCP).

Based on the MIC results, the majority of *A. baumannii* isolates were resistant to both tested antibiotics, with 92 out of 102 strains (90.19%) exhibiting a ciprofloxacin MIC ≥ 4 μg/mL and a levofloxacin MIC ≥ 8 μg/mL, indicating a high level of resistance. Specifically, 90.19% (92/102) of the strains were resistant to ciprofloxacin, 5.88% (6/102) were classified as intermediate, and 3.92% (4/102) of the isolates were susceptible. In contrast, 84.31% (86/102) of the isolates were resistant to levofloxacin, 3.92% (4/102) were intermediate, and 11.76% (12/102) were susceptible. Changes in resistance patterns were observed with the addition of CCCP. For ciprofloxacin + CCCP, 83.83% (85/102) were resistant, 8.82% (9/102) were intermediate, and 7.84% (8/102) were susceptible. For levofloxacin + CCCP, 75.49% (77/102) were resistant, 9.80% (10/102) were intermediate, and 14.71% (15/102) were susceptible (Figure 2 and Table 2).

Our comparative analysis showed that the addition of CCCP resulted in a reduction in the MIC of both ciprofloxacin and levofloxacin. A significant reduction was defined as a 4-fold or greater reduction in MIC. For ciprofloxacin in combination with CCCP, 17.64% (18/102) of isolates showed a significant reduction of 4-fold or greater, 53.92% (55/102) showed a reduction of 2-fold or less, and 28.43% (29/102) showed no change in MIC. For levofloxacin with CCCP, 12.74% (13/102) of the strains showed a significant reduction in MIC, 47.05% (48/102) showed a reduction of less than 2-fold, and 39.21% (40/102) showed no change. Of note, one isolate showed an increase in MIC.

### 3.7 The effect of fluoroquinolone antibiotics and CCCP on the expression of efflux pumps encoding genes

**FIGURE 3:**
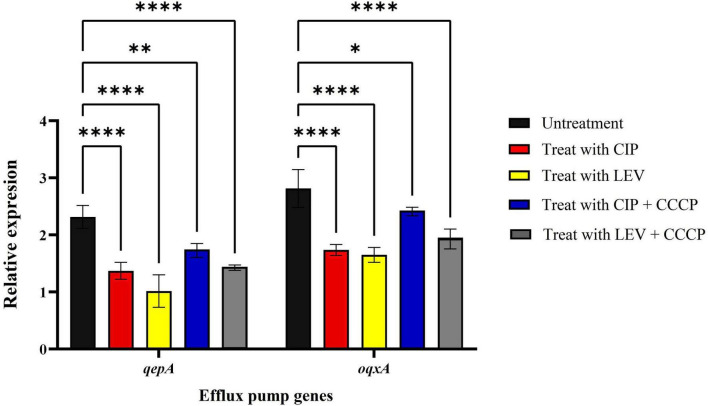
Real-time PCR analysis of qepA and oqxA gene expression in *A. baumannii* isolates. CIP, ciprofloxacin; LEV, levofloxacin; CCCP, inhibidor carbonyl cyanide 3-chlorophenylhydrazone; mean ± SD, *n = 3*, *: *P* < 0.05, **: *P* < 0.01, ****: *P* < 0.0001).

The expression levels of the *qepA* and *oqxA* genes in *A. baumannii* isolates were evaluated via real-time PCR following treatment with ciprofloxacin and levofloxacin, both with and without CCCP, at sub-MIC concentrations. The results showed that the addition of ciprofloxacin and levofloxacin, in combination with CCCP at sub-MIC concentrations, significantly decreased the expression of both *qepA* and *oqxA* genes, with notable reductions compared to the control group (Figure 3).

## 4 Discussion

In 2019, the World Health Organization (WHO) ranked antibiotic resistance among the top ten threats to public health globally, emphasizing its serious implications (31). One of the key pathogens contributing to this issue is *A. baumannii*, a gram-negative coccobacillus that is commonly associated with hospital-acquired infections. This bacterium’s ability to survive for long durations in healthcare environments, along with its resistance to multiple antibiotic classes, plays a significant role in the occurrence of outbreaks. Our study highlights this concerning situation, as we found high levels of antibiotic resistance among 102 *A. baumannii* isolates from Hamadan Regional Hospital in Iran, with 87 identified as MDR.

Fluoroquinolones has been considered as a viable treatment option when *A. baumannii* isolates are sensitive to it, offering a potentially preferable alternative to carbapenems in such cases (32). However, many *A. baumannii* strains resistant to ciprofloxacin also exhibit resistance to various other antibiotic classes. Consequently, alternative treatment strategies, such as carbapenem-sulbactam combinations, colistin, or tetracyclines, are often necessary. Assessing ciprofloxacin resistance is crucial in guiding the appropriate selection of therapeutic interventions for infections caused by *A. baumannii* (33, 34). A significant mechanism contributing to resistance against fluoroquinolones, including ciprofloxacin, and other antibiotics like tetracycline, involves the AdeABC efflux pump system. These efflux pumps actively remove various compounds, including antimicrobial agents, from bacterial cells, thereby facilitating resistance (35, 36). The overexpression of these chromosomal efflux systems, particularly in gram-negative bacteria, is a common driver of multidrug resistance and is associated with the RNA superfamily (37).

Our study revealed a notable presence of PMQR genes among *A. baumannii* isolates, with the *qnrS* gene detected in 77.45% of isolates, *oqxA* in 73.52%, *qnrA* in 72.54%, *oqxB* in 34.31%, and *qepA* in 11.76%. Interestingly, none of the isolates harbored the *qnrB* or *qnrC* genes. These findings are largely in line with those of Mohammed et al., who also reported high rates of PMQR genes in ciprofloxacin-resistant isolates (38). In their study, *qnrA* was found in 66.27% of isolates, *qnrS* in 70.93%, *oqxA* in 73.25%, and *oqxB* in 39.53%, with no detection of *qepA* or *qnrB*. Our study showed slightly higher prevalence rates for *qnrA* and *qnrS*, while the occurrence of *oqxB* was lower. These variations might reflect differences in resistance gene distribution across different regions or healthcare environments. In comparison, Ali et al. reported that 48.3% of *A. baumannii* isolates harbored one or more PMQR genes, a somewhat lower rate than that observed in our study (39). Yang’s study, on the other hand, found a much lower prevalence of PMQR genes among *A. baumannii* isolates. Only 7.7% of isolates carried *qnrB*, and 2.6% carried *qnrS*, highlighting a stark contrast to our findings. This significant difference could be due to regional variations in the spread of resistance genes or differences in the clinical settings from which the isolates were obtained (40). The lower detection rates in Yang’s study underscore the importance of considering geographic and environmental factors when evaluating antimicrobial resistance patterns.

Our PCR-RFLP analysis revealed that among the 102 *A. baumannii* isolates, 38 had mutations in the *gyrA* gene, 34 had mutations in the *parC* gene, and 22 exhibited mutations in both genes. These findings are consistent with those of Tawfick and El-Borhamy, who reported single mutations in either *gyrA* or *parC* in 11 isolates and dual mutations in the QRDRs of both genes in 38 isolates (41). However, our results differ from those of Zaki et al., who found that all ciprofloxacin-resistant *A. baumannii* strains carried mutations in either *gyrA*, *parC*, or both, with dual mutations being the most common (85.5%), and only 5% of strains showing mutations in a single gene (42).

It is well-established that mutations in both *gyrA* and *parC* are generally required for *A. baumannii* to develop high-level resistance to ciprofloxacin (43). In our study, however, we identified 12 ciprofloxacin-resistant and 17 levofloxacin-resistant isolates with single mutations in either *gyrA* or *parC*, yet these isolates still exhibited elevated MICs (≥ 128 μg/mL). This suggests that even single mutations in these genes may contribute significantly to resistance to ciprofloxacin and levofloxacin, which is consistent with the findings of Elshahat et al. (44).

Additionally, our study demonstrated that isolates harboring dual mutations in both *gyrA* and *parC* genes had notably higher MIC values. All such isolates had MICs exceeding 32 μg/mL for ciprofloxacin and 64 μg/mL for levofloxacin, with the majority of double-mutant isolates showing MICs above 128 μg/mL. These results are in line with studies by Ardebili et al. and Taha et al., who reported that strains with mutations in both *gyrA* and *parC* exhibited much higher resistance to ciprofloxacin compared to those with mutations in just one gene (28, 45).

The use of efflux pump inhibitors represents a significant advancement in combating antibiotic resistance (46). This study examined the effect of CCCP on enhancing the susceptibility of *A. baumannii* to ciprofloxacin and levofloxacin. Notably, following the addition of CCCP, 6.38% of the isolates that were previously resistant to ciprofloxacin became sensitive, while 8.82% of the levofloxacin-resistant isolates also showed increased susceptibility. Previous studies have shown that efflux pump inhibitors can significantly reduce multidrug resistance rates in *A. baumannii* and other Gram-negative bacteria. For instance, in the work of Carbonel et al., the addition of CCCP led to 6.38% of imipenem-resistant isolates becoming sensitive to cefepime, with a total of 17.02% of isolates demonstrating increased susceptibility (20). Similarly, Lin et al. reported comparable enhancements in ciprofloxacin susceptibility when CCCP was used (47). Our findings further revealed that 17.64% of isolates exhibited a 4-fold or greater reduction in the MIC for ciprofloxacin, while 12.74 % of isolates showed a similar reduction for levofloxacin. Approximately half of the strains demonstrated a 2-fold dilution reduction in MIC for both antibiotics. Notably, only one strain experienced a 2-fold increase in MIC for levofloxacin. Although our findings are encouraging, other research has reported even greater decreases in MIC values when using efflux pump inhibitors. Gholami et al. showed that the use of Phenylalanine-Arginine β-Naphthylamide led to a 4- to 64-fold decrease in MIC in 96.60% of the 60 *A. baumannii* isolates examined (29). Additionally, Ardebili et al. observed that the susceptibility of *A. baumannii* to ciprofloxacin improved significantly with CCCP treatment, resulting in MIC reductions of 2–64 dilutions for 86.1% of isolates (48). Furthermore, Rajamohan et al. reported that the addition of CCCP caused a 2- to 12-fold decrease in the MIC of various biocides compared to their initial MIC levels (49).

In our study, the expression levels of *qepA* and *oqxA* were evaluated under different conditions, including treatment with ciprofloxacin and levofloxacin, both alone and in combination with CCCP. Our results showed that when CCCP was combined with these antibiotics, it reduced the expression of *qepA* and *oqxA*. However, the most significant reduction in gene expression occurred when the antibiotics were used alone, indicating that the presence of CCCP partially counteracted this effect. Previous studies have demonstrated that CCCP, as an efflux pump inhibitor, influences gene expression in various bacterial species. Rafiei et al. reported that CCCP significantly decreased *adeB* efflux pump gene expression in *A. baumannii* when combined with tigecycline, suggesting its potential in modulating resistance across different antibiotics (50). Similarly, Baron et al. found that CCCP reduced *mcr-1* expression in Gram-negative bacteria, a gene linked to colistin resistance (51). One possible explanation for our findings is that CCCP, by disrupting the proton motive force, alters bacterial stress responses, leading to regulatory shifts in efflux pump gene expression (52). While CCCP is expected to inhibit efflux activity, the partial reversal of antibiotic-induced gene suppression may indicate a compensatory bacterial mechanism aimed at maintaining efflux function despite the inhibitor’s effects. Further research is needed to clarify these regulatory interactions and their implications for resistance management.

## 5 Conclusion

The high prevalence of multidrug-resistant *A. baumannii* isolates in our regional hospital highlights the urgent need for effective antimicrobial strategies. Our findings underscore the significant role of PMQR genes and mutations in *gyrA* and *parC* in driving fluoroquinolone resistance. Although efflux pump inhibitors like CCCP show promise in enhancing antibiotic susceptibility, persistent resistance remains a major challenge. To translate these findings into clinical practice, further research is essential. While this study provides valuable insights, certain limitations must be acknowledged. Conducted in a single regional hospital, the findings may not be fully generalizable to other healthcare settings. Although the sample size was adequate for meaningful analysis, larger multicenter studies would improve the robustness of these conclusions. Additionally, our study primarily focused on genetic resistance determinants; functional assays evaluating efflux pump activity and other resistance mechanisms could further clarify the observed resistance patterns. Future research should prioritize clinical trials assessing the efficacy and safety of CCCP and other efflux pump inhibitors in combination with antibiotics. Moreover, given the clinical importance of antibiotics such as ampicillin-sulbactam and meropenem in the treatment of *A. baumannii* infections, future studies could explore the potential role of efflux pump inhibitors like CCCP as adjunctive agents to enhance the efficacy of these therapies. Investigating such combinations may open new avenues for overcoming resistance and improving patient outcomes in the face of limited treatment options. Investigating such combinations may open new avenues for overcoming resistance and improving patient outcomes in the face of limited treatment options. Furthermore, investigating novel efflux pump inhibitors and alternative combination therapies may offer promising treatment options for *A. baumannii* infections. Continued surveillance of resistance patterns and the development of targeted therapeutic strategies remain essential to combat this growing public health threat.

## Data Availability

The original contributions presented in this study are included in this article/Supplementary material (Supplementary Table 1), further inquiries can be directed to the corresponding author.
